# Perinatal mortality by gestational week and size at birth in singleton pregnancies at and beyond term: a nationwide population-based cohort study

**DOI:** 10.1186/1471-2393-14-172

**Published:** 2014-05-22

**Authors:** Nils-Halvdan Morken, Kari Klungsøyr, Rolv Skjaerven

**Affiliations:** 1 Department of Global Public Health and Primary Care, University of Bergen, Bergen, Norway; 2 Department of Clinical Sciences, University of Bergen, Bergen, Norway; 3 Department of Obstetrics and Gynecology, Haukeland University Hospital, Bergen, Norway; 4 Medical Birth Registry of Norway, Norwegian Institute of Public Health, Bergen, Norway

**Keywords:** Perinatal mortality, Stillbirth, Small-for-gestational-age (SGA), Term, Post-term

## Abstract

**Background:**

Whether gestational age per se increases perinatal mortality in post-term pregnancy is unclear. We aimed at assessing gestational week specific perinatal mortality in small-for-gestational-age (SGA) and non-SGA term and post-term gestations, and specifically to evaluate whether the relation between post-term gestation and perinatal mortality differed before and after ultrasound was introduced as the standard method of gestational age estimation.

**Methods:**

A population-based cohort study, using data from the Medical Birth Registry of Norway (MBRN), 1967–2006, was designed. Singleton births at 37 through 44 gestational weeks (n = 1 855 682), excluding preeclampsia, diabetes and fetal anomalies, were included. Odds ratios (OR) with 95% confidence intervals (CI) for perinatal mortality and stillbirth in SGA and non-SGA births by gestational week were calculated.

**Results:**

SGA infants judged post-term by LMP had significantly higher perinatal mortality than post-term non-SGA infants at 40 weeks, independent of time period (highest during 1999–2006 [OR 9.8, 95% CI: 5.7-17.0]). When comparing years before (1967–1986) versus after (1987–2006) ultrasound was introduced, there was no decrease in the excess mortality for post-term SGA versus non-SGA births (ORs from 6.1 [95% CI: 5.2-7.1] to 6.7 [5.2-8.5]), while mortality at 40 weeks decreased significantly (ORs from 4.6, [4.0-5.3] to 3.2 [2.5-3.9]). When assessing stillbirth risk (1999–2006), more than 40% of SGA stillbirths (11/26) judged to be ≥41 weeks by LMP were shifted to lower gestational ages using ultrasound estimation.

**Conclusions:**

Mortality risk in post-term infants was strongly associated with growth restriction. Such infants may erroneously be judged younger than they are when using ultrasound estimation, so that the routine assessment for fetal wellbeing in the prolonged gestation may be given too late.

## Background

Post-term pregnancy is defined by the World Health Organization and the International Federation of Obstetrics and Gynecology as a pregnancy proceeding to and beyond 294 days of gestation i.e. 42 weeks + 0 days [1-3]. The term prolonged pregnancy has commonly been used about pregnancies proceeding to or beyond 287 days of gestation, corresponding to 41 weeks + 0 days [4]. Both conditions have been associated with numerous maternal and neonatal adverse outcomes [4-10].

When analyzing implications of post-term pregnancy, the method of gestational age estimation is crucial. It is known that ultrasound based estimation of gestational age gives more precise results than estimation based on menstrual dates [11-15]. However, it is also known that ultrasound based estimation tends to shift the entire gestational age distribution towards younger ages [16], probably due to the standards applied for ultrasound measurements at around 18 weeks (the most common time for estimation of due dates). This leads to a decrease in post-term and an increase in preterm gestations when compared to estimations based on menstrual dates [16,17]. This is particularly a problem for infants that are growth restricted, some of which will be growth restricted already at around 18 weeks [18], and thus may be given a “younger” age than they actually have.

Earlier studies on the relation between post-term gestation and perinatal outcome have used last menstrual period (LMP) as basis for gestational age estimation, often because they were done before ultrasound based estimation was an established and well proven standard in clinical practice, or such data were not available [7-9,19-21]. Since ultrasound was introduced in the late 1970s, it quickly replaced last menstrual periods as the standard estimation of gestational age in clinical practice, and thus post-term pregnancies were defined based on ultrasound derived dates.

We hypothesized that death risk in post-term pregnancies is strongly associated with being small-for-gestational age (SGA) and that the implementation of ultrasound-based estimates of gestational age may have had a negative impact especially for the growth restricted infants being assessed as post-term later than they should. The main purpose of the current study was to assess the risk of perinatal death in SGA and non-SGA term and post-term gestations by gestational week, and specifically to evaluate whether the relation between post-term gestation and perinatal mortality was different in the time period before and after ultrasound was introduced as the standard method of gestational age estimation. We also performed analyses in a subset of the data where gestational age from both ultrasound and menstrual date estimations were registered (1999–2006). In this period, we also assessed stillbirth risk separately, using ongoing pregnancies as the risk population (fetus at risk approach).

## Methods

### Data source

A population-based cohort study was designed using data from the Medical Birth Registry of Norway (MBRN) from 1967 to 2006. The register was established in 1967 by the Directorate of Health and was the first national medical birth registry in the world. It is based on compulsory notification of all live births and stillbirths from 16 weeks (since 2002, from 12 weeks) of gestation. A standardized notification form is used to collect data on demographic variables, maternal health before and during pregnancy, previous reproductive history, complications during pregnancy and delivery and pregnancy outcomes. This notification form was almost unchanged from 1967 until 1999. Beginning in 1999, a new and more detailed form was introduced in which maternal smoking habits and ultrasound based due dates were included. All records in the MBRN are matched with the files of the Central Person Register, to ensure medical notification of every newborn in Norway [22], and to collect dates of deaths.

### Study population

We included singleton pregnancies with gestational ages between 37 weeks +0 days (259 days of gestation) and 43 weeks + 6 days (307 days of gestation), thus excluding preterm births. Proportions of misclassified gestational ages were 0.1% in the term and 0.09% in the post-term populations (birth weight >4 standard deviations above or below their gestational week specific mean). We also excluded pregnancies where mothers were registered with preeclampsia, gestational and pre-gestational diabetes, as well as pregnancies where the delivered infant was registered with congenital anomalies. In data from 1999–2006, we excluded daily smoking mothers from the main analyses. However, to assess the impact of adjusting for maternal smoking habits, we did a separate analysis with smoking mothers included (total n = 269 628).

### Definitions and statistical analyses

The following sources are available to estimate gestational age in the MBRN: 1) LMP (registered from 1967) and 2) expected date of parturition according to ultrasound measurements (registered from 1999). Ultrasound based estimation of gestational age was officially introduced to all pregnant women in Norway in 1986 after recommendation from a consensus panel, and very rapidly replaced estimation based on LMPs. The vast majority of obstetric departments in our public health care system (used by nearly all pregnant Norwegian women) used the same standard formula to estimate gestational age by ultrasound during the present study period, based on bi-parietal-diameter (BPD) measurements in the second trimester [23]. When evaluating the implication of the change from menstrual-based to ultrasound-based due dates on the relation between post-term gestation and perinatal mortality, we divided the study period into two: 1967–86 and 1987–2006, and compared the relation between post-term gestation (based on LMP) and perinatal mortality in these two periods. LMP based gestational age was further used for main analyses and time trends, but in a subset of data from 1999–2006 we used ultrasound based gestational age in addition, to compare results using the different gestational age estimations.

We defined post-term pregnancy in accordance with the recommended, standardized and internationally endorsed definition of a pregnancy lasting ≥294 days (42 weeks + 0 days) of gestation. In addition we estimated risks per gestational week from 37 to 42 +.

Perinatal death was defined as stillbirth or death within seven days of life. SGA was defined as infant birth weight by gestational age below the 10th percentile according to a national standard based on data from the MBRN [24]. This criterion is in accordance with common clinical practice [24].

Odds ratios (OR) with 95% confidence intervals (CI) were obtained using logistic regression analyses (IBM SPSS Statistics 19.0 SPSS Inc, Chicago, Illinois, http://www.spss.com). Pregnancies were categorized according to completed gestational weeks into six strata from 37 weeks to ≥42 weeks. Within each week, we further categorized births as SGA or non-SGA. Non-SGA at 40 weeks was used as the reference category in all analyses. The following variables were evaluated as possible confounders, and adjusted for in the logistic regression analyses: Parity (nulliparous or multiparous), maternal age (<20, 20–24, 25–29, 30–34 and 35+ years) and fetal sex. LMP data were analyzed in four separate time periods; 1967–1976, 1977–1986, 1987–1998 and 1999–2006, where reference groups were non-SGA births at 40 weeks in the specific time period. When analyzing the two periods 1967–1986 versus 1987–2006, we also adjusted for time period using two 10-year groups within each main period, and likewise, when analyzing the total material we used four 10-year periods in the logistic regression analyses. In a separate analysis for the years 1999–2006 we also included maternal smoking habits as a confounder (daily smoking yes/no).

Since our main outcome was gestational week specific perinatal mortality, and approximately 2/3 of perinatal death are stillbirths, we also estimated the risk of stillbirth by gestational week separately, using the fetus at risk approach, where ongoing pregnancies at each week constituted the risk population. In order to compare ultrasound and LMP-based estimation of gestational age, these calculations were done for the period 1999–2006.

The MBRN approved the study and provided the data for this analysis. The study was based on anonymous data and was thus exempt from ethical institutional review board approval according to Norwegian legislation.

## Results

In the total cohort of LMP dated pregnancies during 1967 to 2006 (n = 1 855 682), 6 308 perinatal deaths were registered, giving a perinatal mortality risk of 0.34%. For the period 1999 to 2006, where both menstrual and ultrasound based estimation of gestational age was available (n = 234 719), the risk was 0.18%, (412 perinatal deaths; 77 early neonatal and 335 stillbirths).

### LMP-based gestational age 1967–2006

Gestational age specific perinatal death in non-SGA and SGA infants when using LMP based gestational age is reported in Tables 1 and 2, showing results for the different time periods. Table 3 shows results for the total cohort, 1967–2006. The SGA infants had significantly higher perinatal mortality than the reference group in all gestational weeks, and the perinatal mortality showed an inverse J-pattern with lowest risk at 40 weeks and increasing risks both in the weeks below and above 40. Using non-SGA infants at 40 weeks as reference in all time periods, the highest OR of perinatal death for post-term gestations was found among SGA infants during 1999–2006 (adjusted OR: 9.8, 95% CI: 5.7-17.0). The post-term non-SGA infants also had a significantly increased risk of perinatal death, again with the strongest association during 1999–2006: adjusted OR 2.0 (1.4-2.7).

**Table 1:** Perinatal mortality for singleton births in Norway 1967–1976 (n = 531 098) and 1977–1986 (n = 416 735) according to LMP-based gestational age and size at birth (small-for-gestational-age [SGA] and non-SGA)

	**Non-SGA**			**SGA**			
**Gestational age in weeks**	**Perinatal deaths n (per 1000)**	**Adjusted OR**^**α**^	**95% CI**	**Perinatal deaths n (per 1000)**	**SGA% of deaths per week**	**Adjusted OR**^**α**^	**95% CI**
**1967–1976**							
**37**	192 (11.4)	4.1	3.5–4.9	213 (89.0)	52.6	35.1	29.5–41.7
**38**	228 (5.6)	2.0	1.7–2.4	226 (40.7)	49.8	15.3	13.0–18.1
**39**	293 (3.1)	1.1	0.96–1.3	236 (18.3)	44.6	6.9	5.8–8.1
**40**	379 (2.7)	1.0	Reference	222 (11.3)	36.9	4.3	3.6–5.1
**41**	327 (3.0)	1.1	0.98–1.3	187 (12.4)	36.4	4.7	4.0–5.6
**≥42**	316 (4.8)	1.8	1.6–2.1	150 (15.1)	32.2	5.8	4.8–7.0
**1977–1986**							
**37**	94 (7.3)	5.4	4.2–7.0	103 (62.0)	52.3	49.0	37.9–63.4
**38**	110 (3.5)	2.6	2.0–3.3	78 (19.0)	41.5	14.4	11.0–19.0
**39**	155 (2.1)	1.5	1.2–1.9	87 (9.3)	36.0	7.1	5.4–9.2
**40**	147 (1.3)	1.0	Reference	100 (7.4)	40.5	5.6	4.4–7.3
**41**	150 (1.7)	1.3	1.01–1.6	92 (8.8)	38.0	6.7	5.2–8.8
**≥42**	124 (2.4)	1.8	1.4–2.3	57 (8.8)	31.5	6.7	4.9–9.1

Pregnancies with preeclampsia, pregestational and gestational diabetes and infants with congenital anomalies were excluded.

^α^Adjustments by logistic regression where the following confounders were included: maternal age (<20, 20–24, 25–29, 30–34 and 35+), parity (para 0, para 1+) and fetal sex.

**Table 2:** Perinatal mortality for singleton births in Norway 1987–1998 (n = 550 911) and 1999–2006 (n = 244 009) according to LMP-based gestational age and size at birth (small-for-gestational-age [SGA] and non-SGA)

	**Non-SGA**			**SGA**			
**Gestational age in weeks**	**Perinatal deaths n (per 1000)**	**Adjusted OR**^**α**^	**95% CI**	**Perinatal deaths n (per 1000)**	**SGA% of deaths per week**	**Adjusted OR**^**α**^	**95% CI**
**1987–1998**							
**37**	120 (6.1)	4.4	3.5–5.5	78 (36.8)	39.4	27.3	20.9–35.6
**38**	103 (2.1)	1.5	1.2–1.9	87 (16.3)	45.8	11.8	9.1–15.2
**39**	166 (1.6)	1.2	0.94–1.4	100 (8.8)	37.6	6.4	5.0–8.1
**40**	201 (1.4)	1.0	Reference	67 (4.4)	25.0	3.2	2.4–4.2
**41**	157 (1.4)	0.99	0.8–1.2	49 (4.3)	23.8	3.1	2.3–4.3
**≥42**	115 (1.7)	1.2	0.97–1.5	63 (9.3)	35.4	6.8	5.1–9.1
**1999–2006**							
**37**	34 (3.7)	3.5	2.3–5.3	13 (16.6)	27.7	15.8	8.7–28.7
**38**	47 (2.0)	1.9	1.3–2.8	21 (11.5)	30.9	10.9	6.7–17.9
**39**	66 (1.4)	1.4	0.97–1.9	29 (8.8)	30.5	8.3	5.4–12.9
**40**	69 (1.0)	1.0	Reference	13 (3.3)	15.9	3.2	1.7–5.7
**41**	69 (1.3)	1.3	0.9–1.8	16 (5.6)	18.8	5.4	3.1–9.3
**≥42**	63 (2.0)	2.0	1.4–2.7	16 (10.2)	20.3	9.8	5.7–17.0

Pregnancies with preeclampsia, pre-gestational and gestational diabetes and infants with congenital anomalies were excluded. For 1999–2006 smoking mothers were also excluded.

^α^Adjustments by logistic regression where the following confounders were included: maternal age (<20, 20–24, 25–29, 30–34 and 35+), parity (para 0, para 1+) and fetal sex.

**Table 3:** Perinatal mortality for singleton births in Norway 1967–2006 (n = 1 855 682) according to LMP-based gestational age and size at birth (small-for-gestational-age [SGA] and non-SGA)

	**Non-SGA**			**SGA**			
**Gestational age in weeks**	**Perinatal deaths n (per 1000)**	**Adjusted OR**^**α**^	**95% CI**	**Perinatal deaths n (per 1000)**	**SGA% of deaths per week**	**Adjusted OR**^**α**^	**95% CI**
**37**	460 (7.3)	4.2	3.8–4.8	418 (55.2)	47.6	32.5	28.8–36.6
**38**	520 (3.4)	2.0	1.8–2.2	424 (23.3)	44.9	13.3	11.9–15.0
**39**	719 (2.1)	1.2	1.1–1.4	472 (12.0)	39.6	6.7	6.0–7.5
**40**	846 (1.7)	1.0	Reference	416 (7.6)	33.0	4.1	3.7–4.6
**41**	741 (1.9)	1.1	1.02–1.2	352 (8.5)	32.3	4.6	4.1–5.2
**≥42**	648 (2.8)	1.7	1.5–1.8	292 (11.3)	31.1	6.1	5.3–7.0

Pregnancies with preeclampsia, pre-gestational and gestational diabetes and infants with congenital anomalies were excluded.

^α^Adjustments by logistic regression where the following confounders were included: maternal age (<20, 20–24, 25–29, 30–34 and 35+), parity (para 0, para 1+), fetal sex and period (1967–1976, 1977–1986, 1987–1998 and 1999–2006).

To assess if size at birth (SGA or non-SGA) modified the association between post-term gestational age and perinatal mortality, interaction analyses between SGA status and post-term gestational age were performed. We compared the perinatal mortality risk at 40 and ≥42 weeks gestation in a sub-set of the data during 1987–2006 (n = 385 277). The p-value for interaction was 0.01 in a multiplicative model. In stratified analyses the OR for perinatal mortality was 3.1 (2.5-4.0) for non-SGA post-term and 4.9 (3.8-6.4) in SGA post-term infants, using non-SGA infants at 40 weeks as the reference. Adjustments were made for maternal age, parity, fetal sex and time period.

### Before versus after ultrasound estimates were introduced

**Figure 1:**
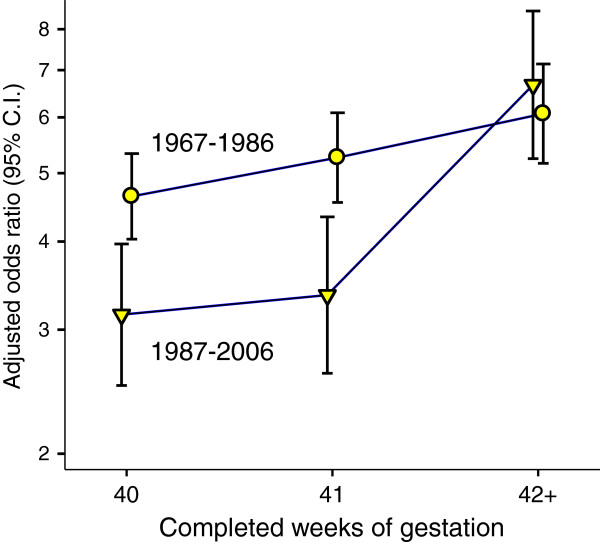
**Odds ratio of perinatal mortality by week of LMP-based gestation in two 20 year periods of the Medical Birth Registry of Norway.** Shown for SGA births and using non-SGA infants at 40 weeks as reference. Estimates were adjusted for maternal age, parity, fetal sex and period.

Figure 1 shows the relation between LMP-based gestational age and adjusted OR of perinatal death for SGA births relative non-SGA births at 40 weeks in two time periods; before (1967–1986) and after (1987–2006) ultrasound was introduced as the standard gestational age estimation method. We see that the increase in excess mortality risk by gestational week from 40 to 42+ is larger in the last than in the first time period. Further, the excess mortality risk, expressed as OR values, for SGA relative non-SGA births at 40 weeks decreased from the first to the second period (4.6 [4.0-5.3] and 3.2 [2.5-3.9], respectively), whereas at 42+ weeks there was no significant change (6.1, [5.2-7.1] and 6.7 [ 5.2-8.5], respectively). We found a significant interaction between time period and LMP based post-term gestation for SGA infants when analyzing the relation between post-term SGA gestation and perinatal mortality, using non-SGA infants at 40 weeks as reference (p < 0.0005, multiplicative model).

### LMP-based gestational age versus ultrasound-based gestational age, 1999–2006

Table 4 shows ORs of perinatal death by gestational week, SGA status and method of gestational age estimation (LMP and ultrasound) in a sub-set of the total cohort (1999–2006). In this table we only show results from pregnancies where both LMP and ultrasound estimation were registered, therefore, data varies slightly from those analyzed in Table 2. The risk of perinatal death was significantly increased in SGA infants at 41 and ≥42 weeks relative non-SGA infants at 40 weeks, independent of gestational age estimation method. However, the strongest association by far was found for LMP-dated infants at ≥42 weeks (adjusted OR: 10.1, 95%CI: 5.8-17.6). For non-SGA infants at 41 and ≥42 weeks, only the post-term infants dated by LMP had significantly increased perinatal mortality (adjusted OR: 1.9, 95% CI: 1.3-2.7).

**Table 4:** Perinatal mortality for singleton births (n = 234 719, of which 412 were perinatal deaths) according to LMP and ultrasound-based gestational age and SGA status in Norway, 1999-2006

	**Gestational age by last menstrual period**					
	**Non-SGA**			**SGA**		
**Gestational age in weeks**	**Perinatal deaths n (per 1000)**	**Unadjusted OR (95% CI)**	**Adjusted OR**^**α **^**(95% CI)**	**Perinatal deaths n (per 1000)**	**Unadjusted OR (95% CI)**	**Adjusted OR**^**α **^**(95% CI)**
**37**	18 (2.39)	2.3 (1.4–3.9)	2.3 (1.3–3.8)	9 (13.8)	13.5 (6.7–27.1)	13.2 (6.5–26.6)
**38**	42 (1.92)	1.9 (1.3 –2.7)	1.8 (1.2–2.7)	17 (9.9)	9.6 (5.7–16.5)	9.5 (5.5–16.2)
**39**	63 (1.40)	1.3 (0.95–1.9)	1.3 (0.95–1.9)	28 (8.8)	8.5 (5.5–13.3)	8.4 (5.4–13.1)
**40**	67 (1.04)	1.0 (Ref.)	1.0 (Ref.)	12 (3.2)	3.1 (1.7–5.7)	3.0 (1.6–5.6)
**41**	67 (1.3)	1.3 (0.9–1.8)	1.3 (0.9–1.8)	13 (4.8)	4.6 (2.5–8.3)	4.5 (2.5–8.3)
**≥42**	60 (2.0)	1.9 (1.3–2.7)	1.9 (1.3–2.7)	16 (10.5)	10.2 (5.9–17.6)	10.1 (5.8–17.6)
	317			95		
	**Gestational age by ultrasound**					
**37**	37 (4.3)	3.6 (2.4–5.3)	3.6 (2.4–5.3)	14 (22.4)	19.0 (10.7–33.6)	18.9 (10.6–33.5)
**38**	44 (1.6)	1.3 (0.93–1.9)	1.3 (0.92–1.9)	28 (14.6)	12.2 (7.9–18.8)	12.0 (7.8–18.5)
**39**	59 (1.1)	0.92 (0.7–1.3)	0.92 (0.7–1.3)	22 (5.7)	4.7 (3.0–7.6)	4.7 (2.9–7.5)
**40**	82 (1.2)	1.0 (Ref.)	1.0 (Ref.)	15 (3.6)	3.0 (1.7–5.1)	2.9 (1.7–5.0)
**41**	61 (1.3)	1.1 (0.8–1.5)	1.1 (0.8–1.5)	11 (4.9)	4.1 (2.2–7.7)	4.0 (2.1–7.4)
**≥42**	34 (1.8)	1.5 (1.0–2.2)	1.5 (0.97–2.2)	5 (7.0)	5.8 (2.4–14.4)	5.5 (2.2–13.7)
	317			95		

Pregnancies with preeclampsia, pregestational and gestational diabetes, infants with congenital anomalies and smoking mothers were excluded.

^α^Adjustments by logistic regression where the following confounders were included: maternal age (<20, 20–24, 25–29, 30–34 and 35+), parity (para 0, para 1+) and fetal sex.

### Maternal smoking

Maternal smoking is a well-known risk factor for SGA and may also affect gestational age and perinatal mortality. We performed a sub-analysis for the years 1999–2006, and included smoking habits (daily smoking yes/no) as a confounder in addition to maternal age, parity and fetal sex, when studying the relation between post-term gestational age and perinatal mortality. When using LMP-based gestational age estimation, the adjusted OR of perinatal death for post-term SGA infants was 8.3 (95% CI: 5.1-13.6) and for post-term non-SGA infants it was 1.8 (95% CI: 1.3-2.4); both relative non-SGA infants at 40 weeks. When ultrasound based gestational age was used, the corresponding ORs were 4.5 (95% CI: 2.0-10.4) and 1.3 (95% CI: 0.9-1.9).

### Stillbirth risk with fetus at risk approach

We repeated analyses for the last time period (1999 to 2006) with gestational age specific stillbirth risk (SGA and non-SGA pregnancies) as outcomes. We compared results using LMP and ultrasound estimation of gestational age. Results are shown in Table 5 where the ongoing pregnancies at each week are the risk population, and non-SGA pregnancies in each week the reference groups. The post-term SGA infants had six to seven times increased risk of stillbirth when compared to post-term non-SGA infants (OR: 7.0 [3.7-13.3] and 6.3 [2.3-16.9]) for LMP-based and ultrasound-based gestational age, respectively. When comparing post-term SGA death to 40 weeks non-SGA, ORs for stillbirth were 20.5 (11.3-37.5) and 13.5 (5.4-33.5) for LMP-based and ultrasound-based gestational age, respectively. There were too few early neonatal deaths (n = 77) to do separate analyses for this group.

**Table 5:** Odds ratio (OR) for stillbirth (335 stillbirths) among 234 719 singleton births by size at birth (small-for-gestational-age [SGA] or non-SGA) and method of gestational age estimation, analyzed with fetus at risk approach, Norway, 1999-2006

**Gestational age by last menstrual period**								**Stillbirths n (%)**
	**Non-SGA**			**SGA**				
**Gestational age in weeks**	**Stillbirths n (%)**	**Ongoing pregnancies (n)**	**Stillbirths per 1000**	**Stillbirths n (%)**	**Ongoing pregnancies (n)**	**Stillbirths Per 1000**	**Adjusted**^**α **^**OR (95% CI)**	
**37**	15 (6.0)	221 151	0.07	8 (9.6)	13 568	0.59	7.4 (3.1–17.7)	23 (6.9)
**38**	37 (14.7)	213 617	0.17	15 (18.1)	12 917	1.16	7.7 (4.1–13.8)	52 (15.5)
**39**	49 (19.5)	191 764	0.26	24 (28.9)	11 207	2.14	8.6 (5.2–14.1)	73 (21.8)
**40**	61(24.2)	146 679	0.42	10 (12.0)	8 029	1.25	3.1 (1.6–6.0)	71 (21.2)
**41**	52 (20.6)	82 300	0.63	13 (15.7)	4 258	3.05	4.8 (2.6–8.9)	65 (19.4)
**≥42**	38 (15.1)	30 689	1.24	13 (15.7)	1 522	8.54	7.0 (3.7–13.3)	51 (15.2)
**Total**	252 (100)	**-**	**-**	83 (100)	**-**	**-**	**-**	335 (100)
**Gestational age by ultrasound**								
**37**	31 (12.3)	221 151	0.14	11 (13.2)	13 568	0.81	6.1 (3.0–12.2)	42 (12.5)
**38**	38 (15.0)	212 557	0.18	25 (30.1)	12 944	1.93	11.3 (6.7–18.8)	63 (18.8)
**39**	48 (19.1)	185 384	0.26	19 (22.9)	11 019	1.72	6.8 (4.0–11.7)	67 (20.0)
**40**	69 (27.4)	132 559	0.52	13 (15.7)	7 156	1.82	3.7 (2.0–6.7)	82 (24.5)
**41**	44 (17.5)	64 786	0.68	10 (12.1)	2 956	3.38	4.7 (2.3–9.3)	54 (16.1)
**≥42**	22 (8.7)	18 779	1.17	5 (6.0)	714	7.00	6.3 (2.3–16.9)	27 (8.1)
**Total**	252 (100)	-	-	83 (100)	-	-	-	335 (100)

Pregnancies with preeclampsia, pregestational and gestational diabetes, infants with congenital anomalies and smoking mothers were excluded.

^α^Non-SGA in the same week was the reference category. Adjustments by logistic regression where the following confounders were included: maternal age (<20, 20–24, 25–29, 30–34 and 35+), parity (para 0, para 1+) and fetal sex. Adjustments only marginally altered the risk estimates.

The number of SGA stillbirths defined as being 41 and ≥42 was 26 when estimation was based on LMP and only 15 when based on ultrasound. Accordingly, 42.3% (11/26) of the prolonged SGA deaths were shifted to lower gestational ages by using ultrasound estimation.

## Discussion

In this study we found strong and consistent associations over time between prolonged and post-term gestational age and perinatal mortality for SGA infants. Further, post-term SGA infants were at significantly higher mortality risk than post-term non-SGA infants. Of particular importance was that the highest excess risk of perinatal death for SGA infants defined as post-term by LMP was found in the last half of the study period, after ultrasound was introduced as a standard estimation method in clinical practice. Assessing stillbirth risk in the last time period, more than 40% of SGA stillbirths were shifted from having prolonged gestation to term weeks when based on ultrasound instead of LMP. Also, for deliveries with due dates postponed by ultrasound during (1999–2006), the OR’s for perinatal death in prolonged and post-term SGA babies (calculated with risk per week) increased from 4.0 and 5.5 to 5.0 and 8.0, respectively (analyses not shown). Routine assessment of fetal wellbeing in the prolonged and post-term gestations would therefore have been missed in these pregnancies.

Our study thus suggests a possible negative impact of changing gestational age estimation method on the relation between post term gestation and mortality risk. The acknowledged shift [16] in gestational age distribution towards younger gestations when using ultrasound measurements at 18 weeks is mostly a problem for growth restricted infants, some of which may be growth restricted also at this early age [18]. These fetuses will mistakenly be judged as younger than they are, and the pregnancies will thus be set up for post-term evaluation too late. At the same time, these growth restricted infants are the exact infants with the highest mortality risk in the post term period. Maternal smoking and fetal sex are among the factors that reduce or affect fetal size in early pregnancy, and have been shown to deflate the risk of post-term delivery when gestational age was based on ultrasound measurements [17]. A recent Swedish study found an increased risk of adverse perinatal outcome among female infants classified as post-term compared with their male counterparts after introduction of ultrasound for estimation of gestational age [25]. Ultrasound gestational age estimation may reduce the total burden of post-term delivery by shifting the entire distribution towards younger gestational ages, but the prize seems to be paid by the small, misclassified fetus.

Our findings of an association between post-term gestation and perinatal mortality for SGA infants are in line with earlier studies [7,19]. However, we also show an interaction between SGA status and post-term gestation in LMP dated pregnancies with significantly higher excess mortality for SGA than non-SGA post-term infants. This suggests that the perinatal mortality risk in the post-term pregnancy is mostly linked to growth restriction rather than to the prolonged gestational age per se.

We also found a significantly increased risk of perinatal death in non-SGA post-term infants when gestational age was based on menstrual dates, whereas mortality was not significantly increased when post-term gestation was based on ultrasound. In a large Californian dataset, Bruckner and colleagues found an increased risk of neonatal mortality in normal-weight prolonged and post-term pregnancies, based on menstrual dates [26]. In the same paper, the authors also expressed the need for more precise estimation of gestational age in these kinds of assessments, and suggested ultrasound estimation [26]. Our results suggest that ultrasound estimation is not uncomplicated, at least when measurements are taken at 18 weeks.

### Strengths and limitations

The most important strength of our study is the large sample size that enabled us to study a rare perinatal outcome by gestational weeks and SGA status. Perinatal death is impossible to study in small scale settings and large datasets are needed, such as that provided by the MBRN.

In this national population-based cohort, selection bias was minimal, as it was based on mandatory reporting of a standardised dataset over a period of 40 years. In a subset of the data we were also able to assess the same set of gestational age specific deaths using two different methods of gestational age estimation at a time when ultrasound was well established, contrary to many other publications on the issue [7-9,19-21].

Information on maternal smoking habits was available from 1999. In a sub analysis for the years 1999–2006 we found that adjusting for smoking habits in addition to maternal age, parity and fetal sex, gave somewhat weaker associations between post-term gestational age and perinatal mortality. However, associations were only marginally affected.

Other studies on the present topic have often merged non-SGA gestations at 37 to 41 weeks into one reference category [19,21]. Our results clearly show that perinatal mortality varies considerably within these five weeks. Due to the large data set, we could use non-SGA infants at 40 weeks as the reference category, which reveals an inverse J-shaped mortality curve even within the term weeks.

### Clinical implications

There has been a debate about the handling of prolonged and post-term pregnancies. The decisions regarding routine induction versus expectant management depends on balancing the effects and acceptability of induction against the effectiveness of intensified fetal surveillance in preventing fetal and infant loss [27]. Inducing delivery before the post-term period is advocated [20,28-30], others suggest that these pregnancies could be managed by intensive fetal surveillance. There is no conclusive evidence that routine induction before 42 gestational weeks improve fetal, maternal or neonatal outcomes compared to expectant management [31,32]. Selective induction is needed only in cases at increased risk while uncomplicated post-term pregnancies are allowed to proceed until spontaneous onset of delivery or induction within the next week. Our data emphasize the importance of identifying the growth-restricted infants in the prolonged and post-term phase of pregnancy due to their increased perinatal mortality risk. However, one of the most important clinical implications of our study is that these growth restricted infants should probably not have their gestational age determined solely by ultrasound.

## Conclusions

Perinatal death risk in prolonged and post-term pregnancies was strongly associated with SGA, independent of time period and method of gestational age estimation. However, the excess mortality risk seen for SGA infants judged to be post-term by LMP has increased after ultrasound estimation has become routine, when compared to non-SGA infants at 40 weeks. This indicates that pregnancies with growth restricted infants may be judged younger than they are when gestational age is estimated by ultrasound. This was further supported by finding that more than 40% of SGA stillbirths judged to be ≥41 weeks by LMP were shifted to lower gestational ages when using ultrasound estimation. Routine assessment of fetal wellbeing in the prolonged and post-term gestations will be missed in these pregnancies.

## Abbreviations

SGA: Small-for-gestational-age; MBRN: Medical Birth Registry of Norway; OR: Odds ratio; LMP: Last menstrual period; CI: Confidence interval.

## Competing interests

The authors declare that they have no competing interests.

## Authors’ contributions

The investigators of this study had full access to all of the data in the study and take responsibility for the integrity of the data and the accuracy of the data analysis. NHM designed the study, performed the analyses of data and was responsible for interpretation of data analysis and completion of the manuscript. KK contributed in interpretation of data analysis and in the completion of the manuscript. RS designed the study, performed analyses, interpreted data, contributed in completion of the manuscript and is guarantor. All authors read and approved the final manuscript.

## Pre-publication history

The pre-publication history for this paper can be accessed here:

http://www.biomedcentral.com/1471-2393/14/172/prepub
